# Chronic Infection With Gastric *Helicobacters* Induces Hepatic Lesions in Mice

**DOI:** 10.1111/hel.70032

**Published:** 2025-04-10

**Authors:** Lornella Seeneevassen, Elodie Sifré, Sadia Khalid, Mathilde Managau, Francis Mégraud, Armelle Ménard, Pierre Dubus, Pirjo Spuul, Christine Varon

**Affiliations:** ^1^ INSERM U1312, Bordeaux Institute of Oncology University of Bordeaux Bordeaux France; ^2^ Department of Chemistry and Biotechnology Tallinn University of Technology Tallinn Estonia; ^3^ Department of Histology and Pathology CHU Bordeaux Bordeaux France

**Keywords:** gastric cancer, *Helicobacter felis*, *Helicobacter pylori*, inflammation, liver, mouse model, steatosis

## Abstract

**Background:**

*Helicobacter pylori* infection is one of the most prevalent chronic bacterial infections worldwide. This bacillus colonizes the human stomach lifelong, where it induces chronic gastritis, evolving in some cases to gastro‐duodenal ulcers, gastric adenocarcinoma, and mucosa‐associated lymphoid tissue lymphoma. *H. pylori* infection has also been associated with extragastric diseases, and clinical data have suggested a role in liver pathogenesis. This retrospective study evaluated the consequences of chronic infection with gastric *Helicobacters* on liver pathogenesis in a mouse experimental model.

**Materials and Methods:**

C57BL6 mice were infected with either *H. felis* (*n* = 12) or five human and mouse‐adapted strains of *H. pylori* (*n* = 77) for one year. Uninfected mice were used as negative controls (*n* = 10). Histopathological analysis of paraffin‐embedded liver tissue sections was performed, and scores were determined in a double‐blind manner for inflammation and steatosis.

**Results:**

Mice infected with *H. felis* and several *H. pylori* strains developed more liver parenchymal inflammation and steatosis, known precursor lesions of liver carcinogenesis, compared to non‐infected mice. The presence of liver lesions was positively correlated with the detection of lesions of the gastric mucosa, more particularly gastric inflammation and metaplasia.

**Conclusion:**

Chronic infection of mice with *H. felis* and *H. pylori* induces liver pathogenesis characterized by parenchymal inflammation and steatosis, which may be associated with the severity of gastric histopathological lesions. Understanding *H. pylori* infection's impact on extragastric lesions could *in fine* help detect and prevent the emergence of other digestive tract‐related diseases.

## 1 Introduction

*Helicobacter pylori* colonizes the stomach of half of the world's population. It has been implicated in a panel of stomach‐related illnesses, including chronic gastritis, gastric and duodenal ulcers, as well as gastric cancers (GC) [1, 2]. Infection with the bacteria is acquired during childhood, and most gastric lesions appear in adulthood, with GC affecting approximately 1% of infected individuals aged approximately 70 years and older.

*H. pylori* infection has also been associated with extragastric diseases, while not as well documented or monitored as gastric ones. Infection by this bacterium has been described in ailments such as Alzheimer's disease, cardiovascular diseases, and idiopathic thrombocytopenic purpura [3, 4, 5, 42, 43]. Moreover, the role of *H. pylori* infection in other digestive diseases like hepatocellular carcinoma (HCC), cirrhosis, fibrosis, pancreatitis [6] as well as colorectal polyps and cancer is also very likely [7, 8].

Indeed, *H. pylori* was detected in 100% of liver tissue from patients with HCC versus 12.5% of liver tissue from patients without HCC, evoking a possible role of the bacteria in the liver carcinogenesis process [9]. In addition, Verhoef et al. [10] found *H. pylori* ‐like DNA in 45% of liver samples from patients with HCC compared to 10% in the control group. This was confirmed by another group showing the presence of *Helicobacter* DNA in 85% of liver samples from patients with HCC in contrast to 33% in the control samples [11]. Other studies also suggested the role of *H. pylori* in the progression of chronic hepatitis, cirrhosis, and HCC in patients positive for both *H. pylori* and hepatitis C virus (HCV). This was due to a higher prevalence of the bacteria in more advanced stages of liver disease such as cirrhosis and HCC (61%–68% and 90%, respectively, compared to 4.2% and 3.5% for control samples and samples from individuals with non‐cirrhotic hepatitis, respectively) [12, 13, 14]. More severe lesions were reported in patients positive for both *H. pylori* and HCV than in patients with only HCV [13]. A meta‐analysis confirmed the higher prevalence of *H. pylori* among patients with chronic hepatitis C relative to controls as well as a 4.48‐fold higher *H. pylori* incidence rate among patients with HCV‐related cirrhosis [15]. Despite these associations between *H. pylori* infection and liver disease observed in clinical human studies, there seems to be very little direct evidence of liver lesions upon gastric *Helicobacter* infection in experimental animal models.

In veterinary clinics, gastric *Helicobacters* were detected in the hepatobiliary system of dogs and cats with hepatic lesions. Analysis of the intrahepatic bile duct of a cat with suppurative cholangitis revealed a semi‐curved bacterium with *Helicobacter*‐like morphology, and molecular analyses confirmed the presence of gastric *Helicobacter* related to *H. nemestrinae* / *H. pylori* [16]. The presence of *H. pylori* was also identified in the liver of stray cats [17]. Boomkers et al. [18] proposed an etiological role of *H. pylori* in feline lymphocytic cholangitis and suggested that cats could be a potential zoonotic reservoir. *ureAB*‐positive gastric *Helicobacter* species were identified in liver samples of dogs presenting hepatic lesions but not in gallbladder samples [19]. Taken together, these data suggest that gastric *Helicobacters*, including *H. pylori* , induce hepatic disorders in infected animals.

In research, the mouse model of chronic infection with *H. pylori* is a pertinent and commonly used model, as it induces chronic gastritis evolving into gastric carcinogenesis in < 1 year [2, 20]. Other studies have described liver lesions following infection with the SS1 mouse‐adapted *H. pylori* strain [21, 22]. *H. pylori* has been successfully detected by immunohistochemistry in liver tissue sections from SS1‐infected mice, and liver inflammation was observed in 3 out of 15 infected mice, suggesting *H. pylori* involvement in hepatitis [23]. However, no quantification data supported these assumptions.

This study, hereby, investigated the impact of *H. pylori* infection on liver histology. C57BL/6 mice were subjected to a 1‐year‐long infection with different human and mouse‐adapted strains of *H. pylori* , as well as with the cat pathogen *H. felis* , as a strong inducer of gastric inflammation and liver histopathology was assessed. These strains were previously reported as being capable of reproducing the *H. pylori* ‐induced gastric carcinogenesis process in this mouse model [24]. The possible association between lesions of the gastric mucosa induced by these gastric *Helicobacters* and lesions of the liver was examined.

## 2 Materials and Methods

### 2.1 Animal Handling

Animal material provided from a previous study approved by the Ethics Committee for Animal Care and Experimentation CEEA 50 in Bordeaux, and experiments were carried out in compliance with the French Ministry of Agriculture (approval number 4608) [24]. Animal experiments were performed on C57BL/6 mice in level 2 animal facilities at the University of Bordeaux. Pathogen‐free 5‐week‐old female mice (free of bacteria including *Helicobacter* species, viruses and parasites), purchased from Charles River Laboratories (L'Arbresle, France), were housed in a humidity‐ and temperature‐controlled 12‐h day/night cycle environment. Mice were fed with a low‐content vitamin diet and water ad libitum. The mice were housed 5 per cage; they all benefited from the same housing conditions with food and water ad libitum [24].

### 2.2 *Helicobacter* Infection

*Helicobacter* strains included *H. felis* strain CS1 (ATCC 49179, A. Labigne, Pasteur Institute, Paris, France) and *H. pylori* strains HPAG1 (L. G. Engstrand, Karolinska Institute, Stockholm, Sweden), SS1, TN2GF4 (Y. Fukuda, Hyogo College of Medicine, Hyogo, Japan), HPARE, and TN2RE (isolated from stomachs of C57BL/6 mice infected with parental strains HPAG1 and TN2GF4, respectively, as previously described [24]). The bacteria were grown on agar plates under microaerobic conditions with 85% N_2_, 5% O_2_, and 10% CO_2_ at 37°C prior to mice infection in PBS at 10 weeks old [24, 25]. *Helicobacter* inoculation was carried out 3 times a week per mouse (every other day) by oral gavage, and PBS alone was used for uninfected controls (10 ≤ *n* ≤ 14 mice per condition) [24, 25].

### 2.3 Tissue Processing for Histological and Molecular Analysis

After mice euthanasia, livers were collected and fixed for 24 h in 3.7% formalin solution (Sigma‐Aldrich, St. Quentin Fallavier, France) before storing in 70% ethanol prior to tissue processing for paraffin embedding following standard procedures. Three micrometer thick sections were then cut from the formalin‐fixed paraffin‐embedded (FFPE) tissues and processed for Hematoxylin/Eosin/Safran (HES) staining as previously described [24].

Relative double‐blinded scoring of liver lesions was carried out on HES‐stained tissue sections. Lesions were scored for tissue inflammation, steatosis, and fibrosis. Liver tissue inflammation was scored separately for parenchymal and perivascular inflammation corresponding to diffuse tissue infiltration by immune cells or accumulation of inflammatory cells around blood vessels, respectively. Scores were attributed as follows: 0: absence of lesions; 1: focal and in a distinct area of the section; 2: multifocal or lesions on 25% to 50% of the section; 3: lesions on > 50% to 80% of the section; 4: lesions on > 80% of the liver section. Total liver lesion scores correspond to the sum of parenchymal inflammation and steatosis scores. Iron deposits were determined after Perls staining according to the manufacturer's recommendations (kit HT20‐1KT, Sigma‐Aldrich), and scored separately for Kupffer cells and hepatocytes using the same score grid as follows determined at 40× magnification objective on 10 microscopic fields: 0: absence of iron deposits; 1: 1 to 5 cells with deposits; 2: 1 cell with deposits per field; 3: at least 2 cells with deposits per field. Total iron deposit scores correspond to the sum of Kupffer cells and hepatocyte scores. Representative images of the different lesions were taken with ×10, ×40, and ×60 magnification objectives using a NIKON Eclipse Ci phase contrast microscope equipped with a NIKON DSRi2 camera and NIS‐BR software (NIKON, Champigny‐sur‐Marne, France).

For quantification of the bacterial load in the liver, real time quantitative PCR experiments were performed on DNA extracted from mouse and liver FFPE tissues on *flaA* gene for *H. felis* and *23S* rRNA gene for *H. pylori* as previously described [24, 26]. The sensitivity of the technique was confirmed on DNA extracted simultaneously from some FFPE stomach tissues of *H. felis* and *H. pylori* ‐infected mice of the same project, which were positive as previously reported [24].

### 2.4 Statistical Analysis

Quantification values represent the mean scores ± SD for 10 ≤ *n* ≤ 14 mice per group. All statistical analyses were performed using GraphPad Prism software v8.0.2 (La Jolla, CA, USA). Statistical differences were calculated using the Mann–Whitney test, Welch's *t*‐test or Student *t*‐test, comparing each infected strain group to the non‐infected control group for each analysis. Tests were chosen according to the Gaussian distribution of samples. Outliers were identified using Jamovi software 2.5.4 (GNU Affero General Public License) and *z*‐score statistics; mice with *z*‐score > 3 were excluded from the analysis. Correlation analysis was carried out using Pearson correlation test, *r* being the correlation coefficient. *p* values < 0.05 were considered as statistically significant. Contingency tables were analyzed using Fisher's exact test to determine the Odds ratio (OR) comparing the odds association between gastric and liver lesions (an OR > 1 shows an association).

## 3 Results

**FIGURE 1:**
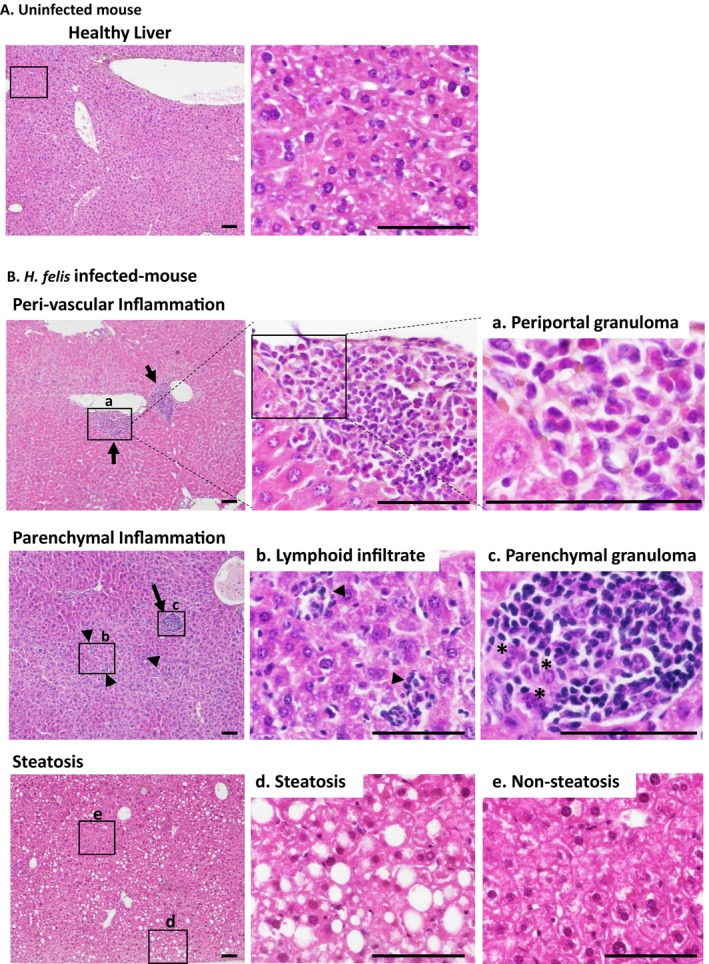
Pathological lesions scored in 1‐year‐long infected and non‐infected mouse livers. Representative images of a non‐inflammatory area in the liver tissue of a non‐infected mouse (A) and of hepatic lesions in *Helicobacter felis*‐infected mice showing perivascular and parenchymal inflammation (B), characterized by focal granulomas (arrows) consisting of an accumulation of various inflammatory cell types such as lymphocytes, plasmocytes, polynuclear cells, and macrophages destroying the hepatocellular cords and areas of diffuse steatosis **d** with a periportal distribution, characterized by intracytoplasmic large vacuoles within the hepatocytes. **a**, periportal polymorphic granuloma composed of macrophages, lymphocytes, plasmocytes, and polymorphonuclear cells; **b**, parenchymal lymphoid infiltrates (arrows heads); **c**, parenchymal granuloma with mainly macrophages and lymphocytes; *residual hepatocellular cells within the granuloma; **e**, non‐steatosis area. All sections were processed for Hematoxylin, Eosin, and Safran staining prior to histological scoring. Scale bars: 100 μm. Box images represent higher magnification of the histology of the healthy liver (**A**, right panel) and the main lesions assessed (**B**, middle and right panels).

The impact of infection with gastric *Helicobacter* species on liver histopathological lesions was evaluated in C57BL/6 wild type female mice. Mice were infected with either different *H. pylori* strains (HPAG1, TN2GF4 and mouse‐adapted strains SS1, HPARE and TN2RE) or *H. felis* strain CS1. After 1 year, stomachs and livers were collected, processed, and analyzed histologically. Stomach lesions were previously described in these mice [24], showing that all strains used were pathogenic and had a primary effect in the stomach. Histopathological evaluation of liver lesions, including inflammation, iron deposit, steatosis, and fibrosis, was carried out (Figure 1).

### 3.1 *H. felis* and *H. pylori* Infection Induce Hepatic Inflammation

**FIGURE 2:**
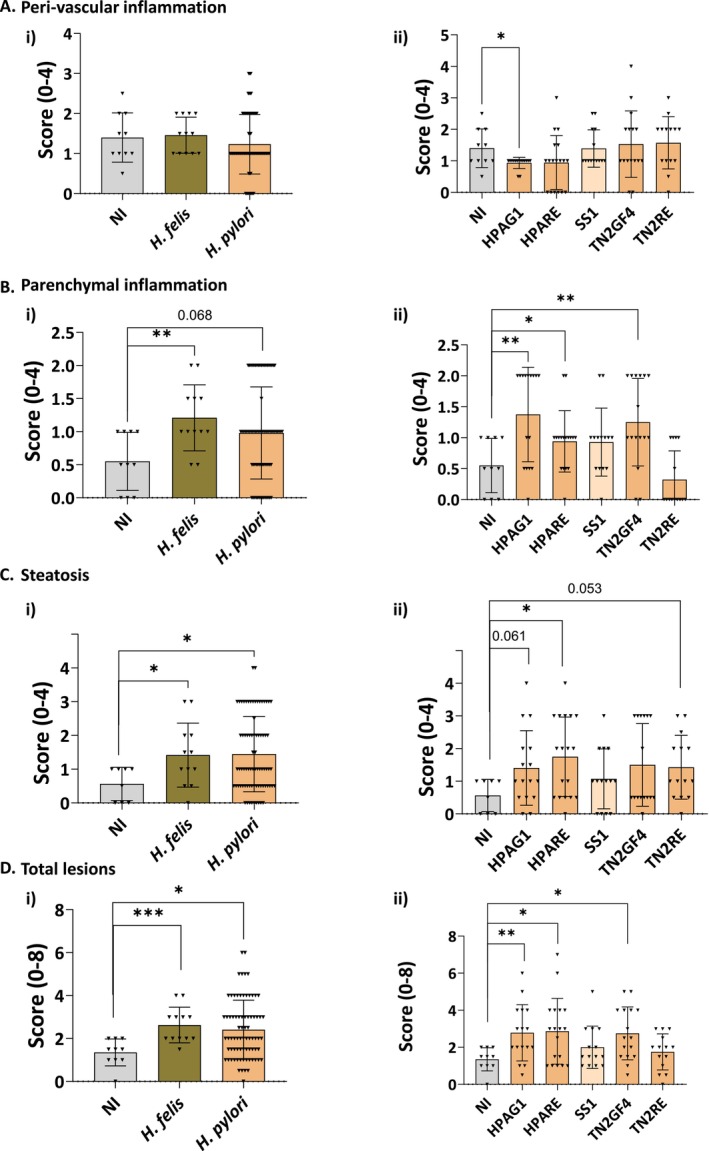
Gastric *Helicobacter* spp. induce parenchymal inflammation and steatosis in 1‐year‐long infected mice. Perivascular inflammation (A), Parenchymal inflammation (B), Steatosis (C) and Total lesions (including parenchymal inflammation + steatosis) (D). Mice were either non‐infected (NI, *n* = 10) or infected with *Helicobacter* spp. *H. felis* (*n* = 12) and *H. pylori* (*n* = 77) (i). *H. pylori* strains used are detailed in (ii) as HPAG1 (*n* = 16), HPARE (*n* = 18), SS1 (*n* = 14), TN2GF4 (*n* = 16) and TN2RE (*n* = 14). Means ± SD are represented. *p < 0.05, **p < 0.005, *** *p* < 0.001 vs. NI; Mann–Whitney, Welch's test, or Student *t‐*test. Each triangle corresponds to a mouse.

Histopathological evaluation of liver lesions is presented in Figure 1. Hepatic inflammation was scored as perivascular (Figures 1B and 2A) or parenchymal with granulomas up to 100 μm in diameter replacing the normal parenchyma (Figures 1B and 2B). They were composed of different cell types such as lymphocytes, plasmocytes, polymorphonuclear cells, and macrophages (Figure 1B, a–c). Both infected and uninfected control mice had some basal perivascular inflammation, which was attributed to aging (Figure 2A). Infection with *Helicobacter* species, especially *H. felis*, showed a significant increase in hepatic parenchymal inflammation and a tendency with *H. pylori* (all strains combined) (Figure 2B). Among the *H. pylori* strains participating significantly in hepatic parenchymal inflammation were HPAG1, TN2GF4, and to a lesser extent HPARE. Iron deposit in hepatocytes and Kupffer cells was evaluated as it is a surrogate marker of hepatic reaction to inflammation. Infection with *Helicobacter* species did not induce any significant increase in total iron deposit (Figure S1), while it correlated with parenchymal inflammation but not with total or perivascular inflammation. These results highlight the fact that chronic infection with *H. felis* and with certain *H. pylori* strains can promote the development of liver parenchymal inflammation.

### 3.2 *H. felis* and *H. pylori* Infection Cause Steatosis but Not Fibrosis

Furthermore, analysis of the liver tissue sections revealed the presence of large intracellular clear vacuoles related to steatosis (Figures 1B and 2C). Relative scoring of these lesions showed a significant increase after infection with both *H. felis* and *H. pylori* . Further assessment of the *H. pylori* strains involved in steatosis demonstrated the significant impact of HPARE to increase steatosis and a clear tendency for two other strains (HPAG1 and TN2RE). No effect was observed on fibrosis, which was not or poorly detected in these 1‐year‐old mice either infected or not (data not shown).

### 3.3 Both *H. felis* and *H. pylori* Infection Causes Hepatic Lesions in Mouse

Finally, the combined analysis of the two informative hepatic lesions, parenchymal inflammation and steatosis (called total liver lesions, Figure 2D) showed that uninfected control mice had unneglectable basal lesions that might have been due to mice aging. *Helicobacter* species infection significantly increased total liver lesions in mice and more particularly *H. felis* , with which we have previously described the greatest effect on gastric damage and carcinogenesis [24] (Figure 2Di). Our analysis also revealed that *cag*PAI+ strains induced more liver damage than the *cag*PAI‐SS1 strain (Figure S2D), with HPAG1, HPARE, and TN2GF4 strains causing significant hepatic lesions (Figure 2Dii).

### 3.4 Correlation Between Gastric and Liver Lesions Following Gastric *Helicobacter* Species Infection

**FIGURE 3:**
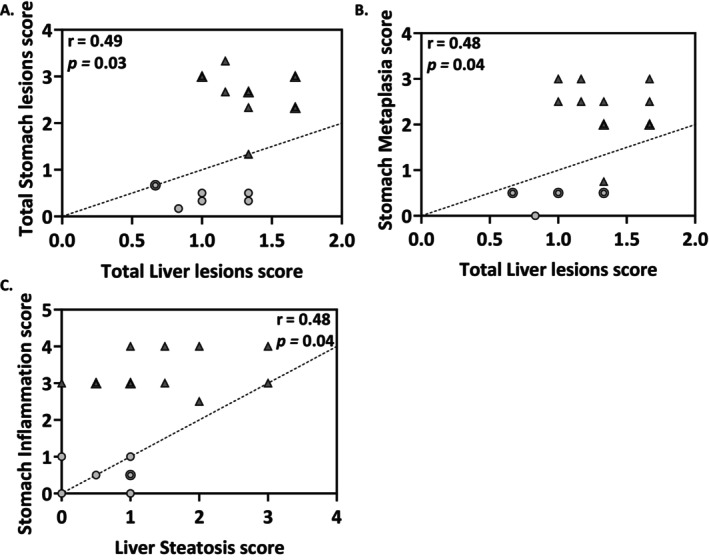
Stomach lesions induced by *H. felis* positively correlate with the appearance of hepatic lesions in 1‐year‐long infected mice. Correlation analysis of Total Liver lesions (corresponding to parenchymal inflammation + steatosis) score with Total Stomach lesions (inflammation, hyperplasia, mucous metaplasia, pseudo‐intestinal metaplasia, dysplasia) score (A) and stomach metaplasia (mucinous metaplasia + pseudo‐intestinal metaplasia) score (B). Correlation analysis of Liver Steatosis score with Stomach Inflammation score (C). Mice were either non‐infected (NI, *n* = 10, gray dots) or infected with *H. felis* (*n* = 12, gray triangles) for 1 year. *r*, correlation coefficient; *p*‐values represented on graphs; Pearson correlation analysis.

Using a multivariate contingency table analysis, we analyzed the association between the presence of hepatic lesions and the previously reported gastric lesions in this model [24]. The strength of the association between the presence of gastric lesions and that of hepatic lesions was ~2.8 fold in all mice combined (*n* = 94 mice, OR = 2.885, 95% confidence interval (CI): 0.5009–13.32, *p* = 0.2434) and of ~4.5 fold in mice infected with *Helicobacter* species (*n* = 87 mice, OR = 4.563, 95% CI: 0.7531–23.21, *p* = 0,1392). This was not linked to the capacity of some *Helicobacter* strains to colonize the liver, as all liver samples were found negative by real‐time quantitative PCR on DNA extracted from FFPE tissues [24, 26]. This result suggests that the observed lesions would not be linked to direct colonization of the liver by the bacteria. We then performed a bivariate linear correlation analysis of data obtained in 1‐year *H. felis* ‐infected mice (*n* = 12 mice), corresponding to the group showing the most lesions compared to the other strains, and the uninfected group showing the least lesions (*n* = 10 mice). We found that the presence of stomach lesions (inflammation, hyperplasia, atrophy, mucinous and pseudo‐intestinal metaplasia, and dysplasia of the gastric mucosa [24]) was positively associated with the development of liver lesions (parenchymal inflammation and steatosis) (*r* = 0.49, *p* = 0.03, Figure 3A). In addition, total liver lesions were significantly associated with gastric metaplasia (*r* = 0.48, *p* = 0.04, Figure 3B), and liver steatosis was significantly associated with gastric inflammation (*r* = 0.48, *p* = 0.04, Figure 3C). Total liver lesions and liver steatosis were not correlated with the body weight of mice (Figure S3). These results suggest that the development of liver parenchymal inflammation and steatosis may be associated with gastric *Helicobacter*‐induced inflammation of the gastric mucosa and the severity of gastric histopathological lesions.

## 4 Discussion

*H. pylori* implication in gastric carcinogenesis is now an undisputed fact. The important question remaining at least partially unanswered is the implication of the bacteria in extragastric digestive organs and more particularly liver pathogenesis.

Indeed, the role of gastric *Helicobacter* species in hepatic lesions has been suggested in some studies describing the presence of the bacterium in liver samples from subjects either presenting HCC or liver lesions [9, 19]. Few studies have evaluated the hepatic lesions that might arise from *Helicobacter* infection of the stomach, and most of them used the mouse‐adapted *H. pylori* strain SS1 [22, 27]. Huang et al. [23] previously suggested that inflammation and steatosis occur in the liver of C57BL/6 mice following chronic infection with the *H. pylori* SS1 strain; however, no quantitative data supported these findings.

We hereby carry out an extensive screening of hepatic lesions following infection with six different strains of gastric *Helicobacters*, including *H. felis* and five strains of *H. pylori* . In a previous study, we showed that all of these strains had the capacity to colonize the stomach and to induce stomach inflammation and pre‐neoplastic lesions leading to gastric carcinogenesis [24]. We show here that these mice also exhibited liver damage in response to *H. felis* and most of the *H. pylori* strains used in this study, corresponding to parenchymal inflammation and steatosis but not fibrosis. The cat pathogen *H. felis* , known for its high pathogenicity in mouse models of gastric infection, induced the most important liver damage. Concerning *H. pylori* , among the five strains used, three induced parenchymal inflammation and three steatosis, independent of their parental (HPAG1, TN2GF4) or mouse‐adapted (HPARE, TN2RE) origin. However, the *H. pylori* SS1 strain had no effect on liver damage, although it was shown to be the second strongest inducer of inflammatory and pre‐neoplastic gastric lesions after *H. felis* [24]. These effects could be associated with some of the main pathogenic factors of *H. pylori* involved in its carcinogenic potential, as all *cag*PAI+, *cag*A+, and *vac*A (s1m1) + *H. pylori* strains (HPAG1, HPARE, TN2GF4 and TN2RE) induced parenchymal inflammation (except TN2RE) and hepatic steatosis (except TN2GF4) contrary to the SS1 strain deficient for both *cag*PAI (incomplete, leading to a nonfunctional T4SS) and active VacA (*vac*A genotype s2m2, leading to a non‐secreted poorly active toxin) which had no significant effect (Figure 2B,C, Table 1, Figure S2). Further studies are necessary to evaluate the involvement of the T4SS and CagA oncoprotein, encoded by the *cag*PAI, and VacA toxin, on liver pathogenesis upon *H. pylori* infection, since both lead to a proinflammatory response and to oncogenic signaling of the target epithelium.

**TABLE 1:** Pathogenicity factors of *H. pylori* strains used in this study [24].

	HPAG1 and HPARE	TN2GF4 and TN2RE	SS1
**Genotype**			
*cag*PAI	+	+	Partially deleted
*cag*A	+	+	+ but non functional
*cag*E	+	+	+ but non functional
*vac*A	s1m1	s1m1	s2m2
**Effect on infected gastric epithelial cells**			
IL8 secretion (proinflammatory)	+	+	−
Cell elongation (epithelial‐mesenchymal transition)	+	+	−
Vacuoles (pore‐forming toxin, pro‐apoptotic)	+	+	−

*Note:* In infected gastric epithelial cells in vitro, *cag*PAI+ *cag*E+ *H. pylori* strains induce a proinflammatory response characterized by IL‐8 secretion, and *cag*A+ strains induce cell elongation, which is reminiscent of an epithelial‐mesenchymal transition occurring during the gastric carcinogenesis process. The s1m1 *vac*A genotypes are responsible for the formation of intracellular vacuoles leading to mitochondrial disturbance and apoptosis. The VacA toxin produced by s2m2 genotype is not pathogenic because it is not secreted by the bacteria. SS1 strain possesses a *cag*PAI but truncated so it encodes for a nonfunctional T4SS [24]. +, positive genotype or positive effect; −, negative effect on gastric epithelial cells in vitro.

This study suggests that the use of *H. pylori* strains possessing a functional T4SS (full *cag*PAI) and an active VacA (s1m1 genotype) could be essential to study liver pathogenesis in mouse models. Indeed, many other factors of *H. pylori* contribute to its pathogenicity, including the large group of outer membrane proteins (OMPs), containing some adhesins (BabA and SabA), adherence‐associated lipoprotein (AlpA/B), and inflammatory OMPs (OipA or HomB), that mediate *H. pylori* binding to the host cells; the ADP‐glycero‐β‐D‐manno‐heptose (ADP‐heptose) metabolite of lipopolysaccharide (LPS); the urease; and the serine protease HtrA, whose single nuclear polymorphism has recently been associated with disruption of the gastric epithelial barrier and gastric cancer [28, 29, 44]. In addition, we have previously reported the formation of podosomes, corresponding to actin‐based subcellular structures, involved in extracellular matrix remodeling in *H. pylori* ‐infected primary mouse hepatocytes in vitro [30] through the release of proinflammatory cytokines such as TGF‐β1 and TNFα. It has also been shown that *H. pylori* promotes TGF‐β1‐related hepatic fibrosis in models of chemically induced liver damage and fibrosis [27]. All of this accounts for the significant need in understanding *H. pylori* pathogenic factors and related molecular mechanisms leading to liver pathogenesis.

This study shows that infection with gastric *Helicobacter* species can lead to liver inflammation and steatosis in mice. Steatosis, also described as the abnormal accumulation of lipids in cells, is one important hepatic lesion that can lead to fatty liver or nonalcoholic fatty liver disease (NAFLD) and finally to nonalcoholic steato‐hepatitis (NASH) which is a type of NAFLD accompanied by inflammation. Our findings correlate with the study from Dogan et al. [31] in which fatty liver was more often diagnosed in *H. pylori* ‐positive patients than in negative ones. Furthermore, other studies show higher *H. pylori* positivity in patients with NAFLD compared to control groups, in line with our results [32, 33]. *H. pylori* infection also increases the risk of developing NAFLD in diabetic patients [34]. Further studies in mice predisposed to develop pathological inflammation and steatosis, such as in response to a high‐fat diet regimen [35], could lead to more severe lesions in response to gastric *Helicobacter* species infection.

Among the mechanisms that could potentially be causing *H. pylori* ‐related inflammation and steatosis is systemic inflammation caused by *H. pylori* infection. Indeed, infection by *H. pylori* causes proinflammatory cytokines (TNFα, IL‐1β, IL‐6 and IL‐8) to be released, which in turn can lead to increased oxidative stress in the liver, disruption of the hepatic cell architecture, necrosis, inflammation, and ballooning of liver cells [36]. Furthermore, *H. pylori* infection also induces insulin resistance (IR) which can have a role in steatosis development since it favors the accumulation of free fatty acids in the liver [37]. Studies have shown that *H. pylori* infection is capable of inhibiting insulin signaling through c‐Jun/miR‐203/SOCS3 signaling pathways. The infection decreases serum leptin levels, thus altering insulin signal transduction and fat metabolism [38]. It causes hypertriglyceridemia and hypercholesterolemia, leading to hepatocyte steatosis [39]. Additionally, some studies show that *H. pylori's* antigen fragments as well as virulence factors (VacA) can be detected in infected livers. In the case of VacA, the patients were found to suffer from mild hypertransaminasemia [36]. *H. pylori* infection has also been associated with increased aminotransferase activity, which reflects liver damage.

Accurate monitoring of caloric intake, body weight, metabolic dysfunction, and insulin resistance, as well as the detection of proinflammatory cytokines and molecular profiles associated with *Helicobacter* in the blood and liver, would provide a better understanding of confounding factors associated with *Helicobacter*‐induced liver injury. Antibiotic eradication therapy would also help determine whether *Helicobacter*‐induced liver injury is reversible, and if so, to what extent.

The microbiota at the intersection of the gut–liver axis could also play a major role in liver pathogenesis. Chronic infection with *H. pylori* is associated with gastric and intestinal dysbiosis and an increased permeability of the gastrointestinal epithelium leading to translocation of bacteria and bacterial components (including cell‐wall components and toxins from gram‐negative bacteria). These pathogen‐associated molecular patterns are recognized by receptors of innate immunity on liver cells, leading to a chronic inflammatory response and to liver damage [40].

This study and the further investigations could contribute to the recognition of infection with gastric *Helicobacter* species such as *H. pylori* not only as a gastric carcinogen but also as a risk factor of liver pathogenesis. Therefore, it shows the importance of taking into account *H. pylori* infection status for patient support and therapy when diagnosed with hepatic diseases. In line with this, a recent study proposed the use of *H. pylori* ‐eradication therapy in patients with NAFLD and compared it to the standard management therapy for NAFLD, which is weight reduction [41]. Though *H. pylori* ‐eradication therapy did not show a better effect than standard management therapy on hepatic steatosis, it was found to improve insulin resistance, one important factor in NAFLD progression that can also be *H. pylori* ‐related. This study also implies that patients with *H. pylori* infection should not only be monitored for gastric‐related diseases and GC but also for hepatic diseases.

## 5 Conclusion

In conclusion, despite the fact that uninfected C57BL/6 mice already presented some basal levels of inflammation and mild steatosis at 1 year, this study shows that chronic infection with gastric *Helicobacter* species, including *H. felis* and most of the *H. pylori* strains tested, has an impact on the development and/or promotion of liver parenchymal inflammation and steatosis. We found that the presence of liver lesions was positively correlated with the presence of gastric lesions. *In fine*, our data combined with those of the literature suggest that chronic infection with gastric *Helicobacter* species, including *H. pylori*, participates in liver pathogenesis and that analyzing *H. pylori* infection status in patients with liver disease and conducting an eradication therapy might be a way of slowing down the progression of the disease and the appearance of its disastrous consequences.

## Author Contributions

All authors have substantially contributed to the manuscript and agreed to the submitted version. C.V., P.D., P.S.: concept, design or methodology. L.S., E.S., S.K., M.M., C.V., P.D., P.S.: acquisition, analysis, interpretation, visualization of data. L.S., E.S., C.V., P.D., A.M., P.S., F.M.: writing, review, and editing. C.V., P.D., F.M.: funding acquisition, resources. C.V.: study supervision and administration.

## Supporting information


Figures S1–S3

## Data Availability

The data that support the findings of this study are available on request from the corresponding author. The data are not publicly available due to privacy or ethical restrictions.
