# Supplementary material for: Chronic Infection With Gastric Helicobacters Induces Hepatic Lesions in Mice
Source: Helicobacter. 2025 Apr 10;30(2):e70032. doi: 10.1111/hel.70032 (PMC11984071; doi:10.1111/hel.70032)

**Supplementary materials**

**Supplementary Figure Legends**

**Supplementary Figure S1. Iron deposits scored in one-year long *Helicobacter-*infected and non-infected mouse livers.** Representative images of Perls staining in a liver tissue section of an *H. pylori* HPAG1-infected mouse with iron deposits in both hepatocytes (arrows) and Kupffer cells (arrow-heads) **(A)** and a non-infected mouse in an area without iron deposit **(B)**. **C**, Relative quantification of total iron deposit corresponding to the sum of iron deposits scores for Kupffer cells and hepatocytes. Means ± SD are represented. NI, non-infected mice (n=8). Mice infected with *H. felis* (n=11) or *H. pylori* strains (n=71) corresponding to HPAG1 (n=16), HPARE (n=17), SS1 (n=11), TN2GF4 (n=14) and TN2RE (n=13) for one year. ******p <* 0.05 *vs.* NI; Mann-Whitney or Student t-test. **D**, Correlation analysis of Total Iron deposit (including Kupffer cells and hepatocytes) scores with scores for parenchymal inflammation, perivascular inflammation or total liver inflammation (corresponding to the sum of both parenchymal and perivascular inflammation). r, correlation coefficient; *p*-values represented on graphs; Pearson correlation analysis.


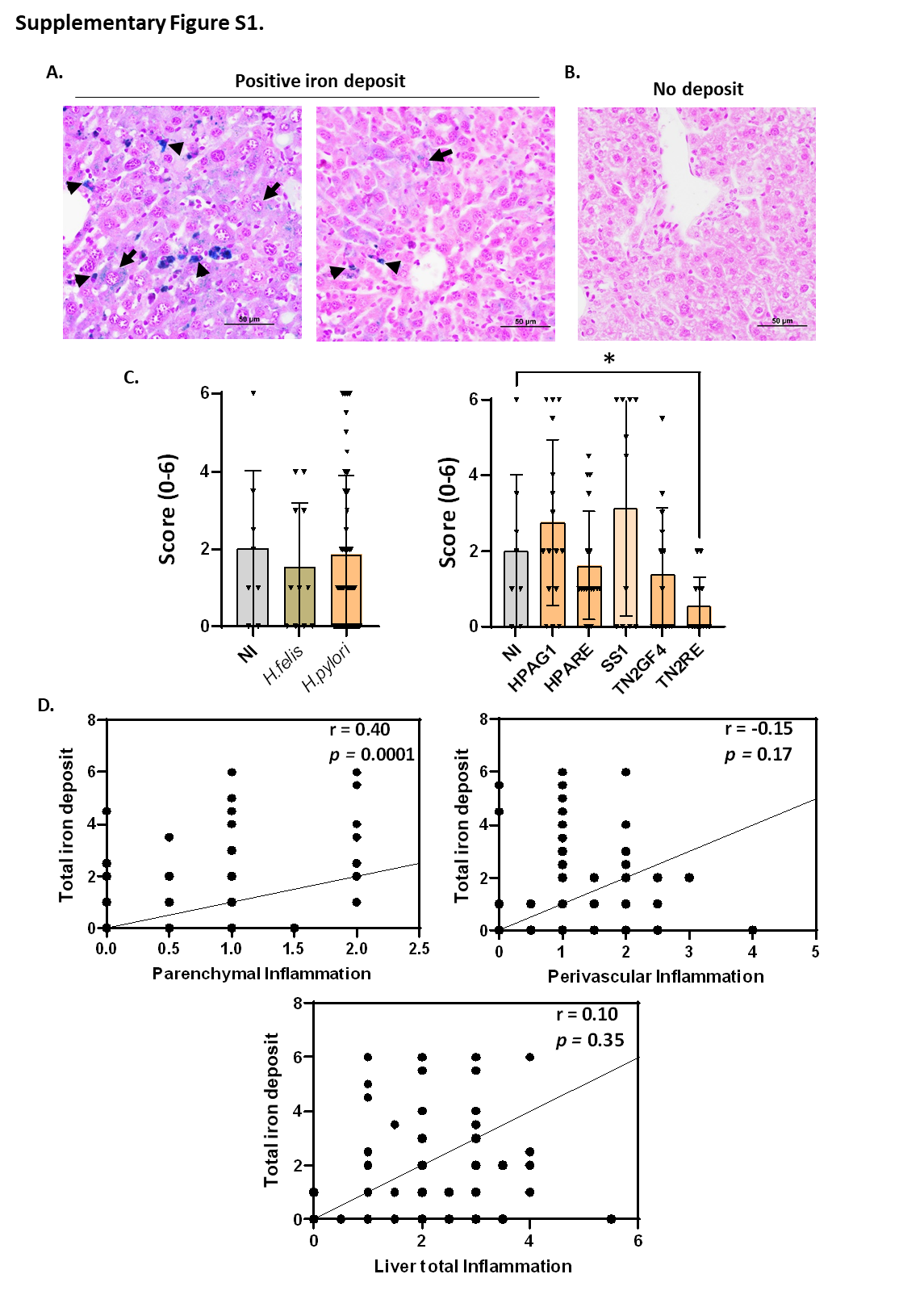


**Supplementary Figure S2. *cag*PAI+ *H. pylori strains* induce more liver damage in one-year long infected mice.** Perivascular inflammation **(A)**, Parenchymal inflammation **(B)**, Steatosis **(C)** and Total lesions (parenchymal inflammation + steatosis, **D)**. Mice were either non-infected (NI, gray bars) or infected with *H. pylori* *cag*PAI- (SS1, light orange bars) or *cag*PAI+ (HPAG1, HPARE, TN2GF4, TN2RE, n=64, dark orange bars) strains. Means ± SD are represented. ******p <* 0.05, *******p <* 0.01 *vs.* NI; Mann-Whitney or Student t-test.


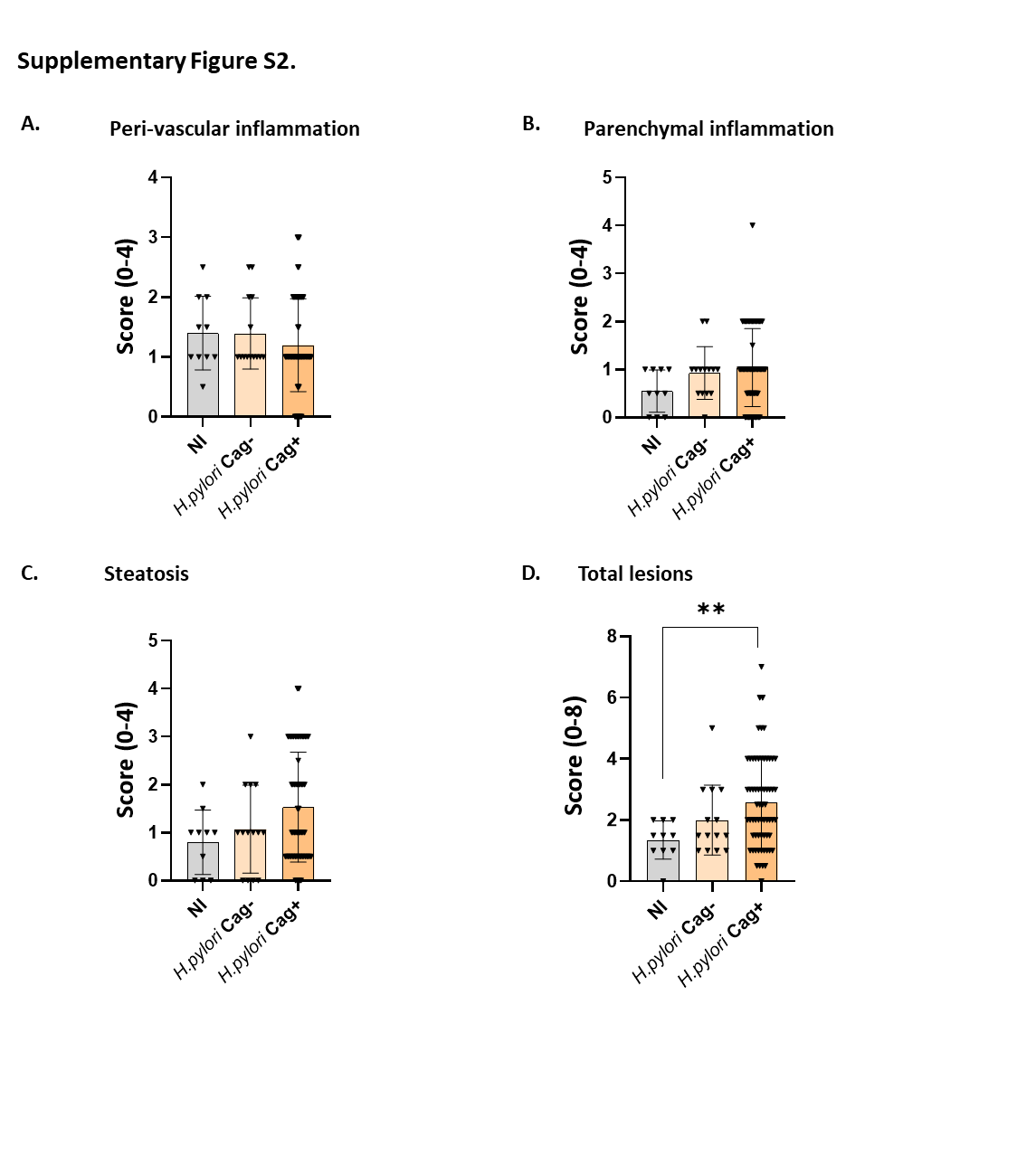


**Supplementary Figure S3. Correlation analysis of mice weight with hepatic lesions in one-year long *Helicobacter-*infected and non-infected mouse livers.** **A**, Correlation analysis of mice weight with scores for liver steatosis (A) or total liver lesions (B) (corresponding to the sum of parenchymal inflammation and steatosis), r, correlation coefficient; *p*-values represented on graphs; Pearson correlation analysis.


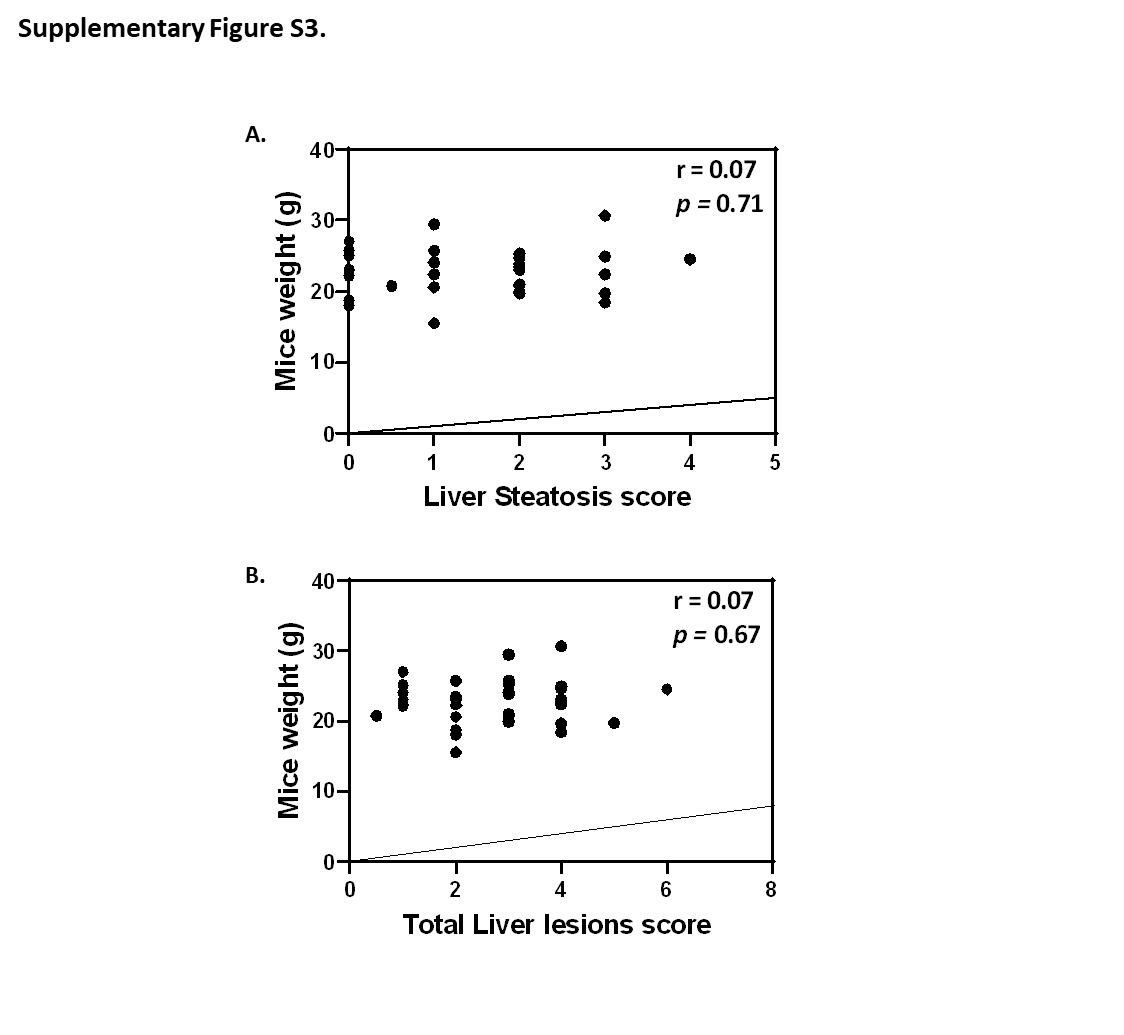

Supplement: Supplementary file 1 — Figures S1–S3 [file HEL-30-e70032-s001.docx]
